# Validation of the Meet-URO score in metastatic clear cell renal cell carcinoma patients receiving second or third-line tyrosine kinase inhibitors-immune checkpoint inhibitors combination therapy

**DOI:** 10.1186/s12967-024-05014-z

**Published:** 2024-03-03

**Authors:** Sike He, Haoyang Liu, Junru Chen, Hao Zeng

**Affiliations:** Department of Urology, Institute of Urology, West China Hospital, Sichuan University, Chengdu, China

## To the editor,

In recent years, the combinations of tyrosine kinase inhibitors (TKIs) and immune checkpoint inhibitors (ICIs) have become the standard treatments for metastatic renal cell carcinoma (mRCC) irrespective of the International Metastatic RCC Database Consortium (IMDC) prognostic groups [1]. Despite their overall efficacy, not all patients achieve satisfactory response and long-term survival benefits [2]. Hence, the identification of biomarkers and prognostic models to select patients for these combination therapies is a crucial issue in clinical practice.

The Meet-URO score is a novel prognostic model incorporating the presence of bone metastases and baseline peripheral-blood neutrophil-to-lymphocyte ratio (NLR) ≥ 3.2 into the IMDC score, which has been shown to have a better prognostic value than the IMDC score in mRCC patients receiving nivolumab [3]. However, whether the Meet-URO score could accurately predict the prognosis in the TKIs-ICIs combination treatment setting remains unclear. This analysis aims to investigate the prognostic value of the Meet-URO score in mRCC patients receiving 2nd and 3rd-line TKIs-ICIs combination treatment, which has not been reported by previous studies.

Baseline and follow-up data of patients with mRCC were obtained retrospectively from West China Hospital. Overall survival (OS) was defined as the time from the start of 2nd or 3rd -line therapy to death from any cause or to the time of last follow-up for survivors, and progression-free survival (PFS) was calculated from 2nd or 3rd -line therapy start to 2nd or 3rd disease progression or death without disease progression and to death or last follow-up visit. Both OS and PFS were assessed by the Kaplan–Meier method. Harrell’s *c*-index was estimated to evaluate the accuracy of the prediction ability of the two score models. The Cox proportional hazard-regression model was used for univariable and multivariable analyses. Hazard ratios (HR) with a 95% confidence interval (CI) were calculated. All the statistical analyses were performed by using R software (*v* 4.1.0). The *p*-value < 0.05 was considered statistically significant.

**Fig. 1:**
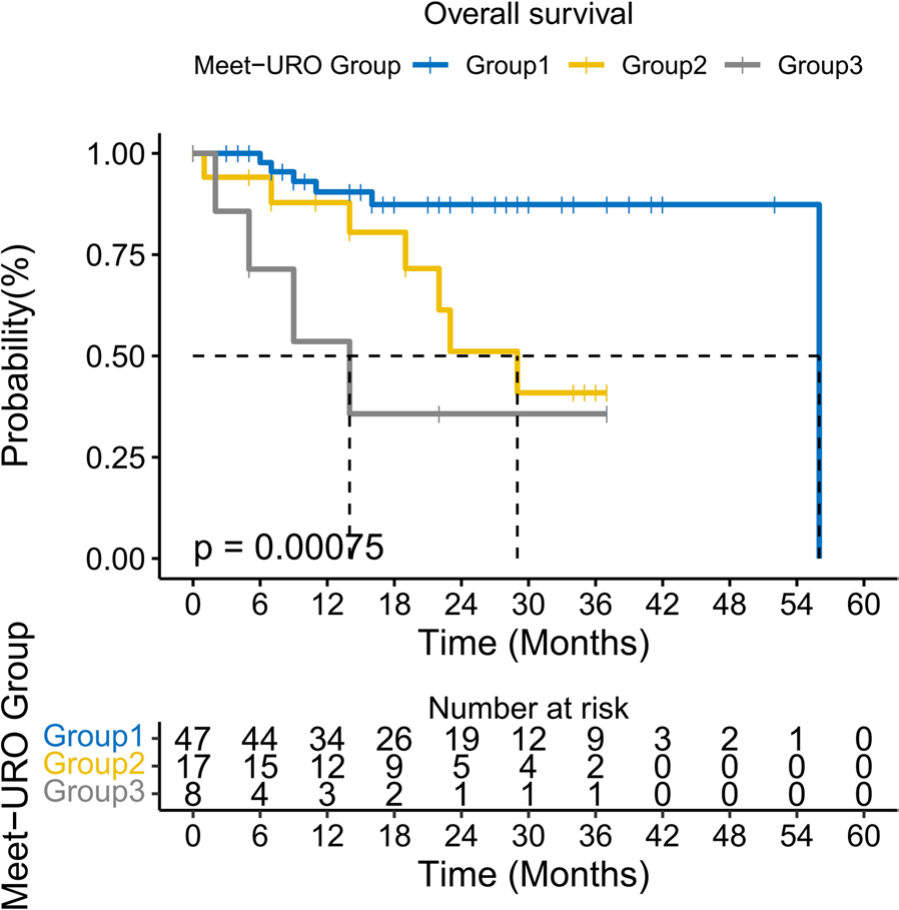
Kaplan–Meier curves for OS according to the Meet-URO score

A total of 72 patients receiving 2nd and 3rd-line TKIs-ICIs combination (2nd-line: 88.89%, 3rd-line: 11.11%) were included in the final study. The detailed baseline information is provided in Table 1. Patients were divided into three groups according to the Meet-URO score (group 1: score 0–3, group 2: score 4–5, and group 3: score 6–8). Patients in group 1 had the most favorable prognosis with a median OS (mOS) of 56 months. Compared to group 1, group 2 (mOS: 29 *vs.* 56 months, p = 0.013) and group 3 were associated with significantly poorer survival outcomes (mOS: 14 months *vs.* 56 months, p < 0.001, Fig. 1). There was no statistically significant difference in PFS between the three groups. Compared to the IMDC score, the Meet-URO score had a higher *c*-index (0.706 *vs*. 0.560), which indicated that the Meet-URO score had a higher discriminative ability than the IMDC score in this setting. The univariate analysis revealed that a higher Meet-URO score correlated with shorter OS. However, pre-treatment nephrectomy was a protective factor for prognosis in the univariate analysis. In the multivariate analysis, the Meet-URO score was the only independent prognosticator for OS (Additional file 2).

**Table 1:** Baseline characteristics of included patients

Characteristics	Total	Meet-URO Group 1 (Score: 0–3)	Meet-URO Group 2 (Score: 4–5)	Meet-URO Group 3 (Score: 6–8)	*p-*value
Sample size (n)	72	47	17	8	
ccRCC (%)	72 (100)	47 (100)	17 (100)	8 (100)	NA
Gender (%)					0.443
Male	61 (15.28)	8 (17.02)	3 (17.65)	0 (0.00)	
Female	11 (84.72)	39 (82.98)	14 (82.35)	8 (100.00)	
Age (median [IQR])	56.50 (52.00, 66.00)	56 (51.50, 66.00)	56.00 (54.00, 63)	62.00 (53.75, 67.50)	0.507
Nephrectomy (%)					0.004
Yes	62 (86.11)	44 (93.62)	14 (82.35)	4 (50.00)	
No	10 (13.89)	3 (6.38)	3 (17.65)	4 (50.00)	
ISUP (%)					0.248
ISUP < 3	18 (25.00)	14 (29.79)	4 (23.53)	0 (0.00)	
ISUP ≥ 3	37 (51.39)	23 (48.94)	10 (58.82)	4 (50.00)	
NA	17 (23.61)	10 (21.28)	3 (17.65)	4 (80.00)	
T stage ≥ 3 (%)					0.337
Yes	24 (33.33)	17 (36.17)	4 (23.53)	3 (37.50)	
No	26 (36.11)	16 (34.04)	9 (52.94)	1 (12.50)	
NA	22 (30.56)	14 (29.79)	4 (23.53)	4 (50.00)	
N stage (%)					0.611
0	48 (66.67)	33 (70.21)	11 (64.71)	4 (50.00)	
1	11 (15.28)	5 (10.64)	4 (23.53)	2 (25.00)	
Nx	3 (4.17)	2 (4.26)	0 (0.00)	1 (12.50)	
NA	10 (13.89)	7 (14.89)	2 (11.76)	1 (12.50)	
M stage (%)					
Synchronous	39 (54.17)	29 (61.70)	8 (47.06)	2 (25.00)	
Metachronous	33 (45.83)	18 (38.30)	9 (52.94)	6 (75.00)	
IMDC group (%)					< 0.001
Favorable	16 (22.22)	16 (34.04)	0 (0.00)	0 (0.00)	
Intermediate	48 (66.67)	31 (65.96)	17 (100.00)	0 (0.00)	
Poor	8 (11.11)	0 (0.00)	0 (0.00)	8 (100.00)	
Bone metastasis (%)					0.435
Yes	13 (18.06)	3 (6.38)	8 (47.06)	2 (25.00)	
No	59 (81.94)	44 (93.62)	9 (52.94)	6 (75.00)	
NLR ≥ 3.2 (%)					0.579
Yes	14 (19.44)	2 (4.26)	9 (52.94)	3 (37.50)	
No	58 (80.56)	45 (95.74)	8 (47.06)	5 (62.50)	
Prior treatment (%)					0.776
TKI	66 (91.67)	44 (93.62)	15 (88.24)	7 (87.50)	
TKI + IO	4 (5.55)	2 (4.26)	1 (5.88)	1 (12.50)	
TKI + mTOR	2 (2.78)	1 (2.12)	1 (5.88)	0 (0.00)	
2nd-line treatment (%)					< 0.001
Yes	64 (88.89)	44 (93.62)	13 (76.47)	7 (87.50)	
No	8 (11.11)	3 (6.38)	4 (23.53)	1 (12.50)	
Based immunotherapy (%)					0.643
NIV	2 (2.78)	2 (4.26)	0 (0.00)	0 (0.00)	
PEM	7 (9.72)	4 (8.51)	2 (11.76)	1 (12.50)	
SIN	39 (54.17)	27 (57.45)	7 (41.18)	5 (62.50)	
TIS	3 (4.17)	3 (6.38)	0 (0.00)	0 (0.00)	
TOR	21 (29.17)	11 (23.40)	8 (47.06)	2 (25.00)	

*ccRCC* clear cell renal cell carcinoma, *IMDC* International Metastatic Renal Cell Carcinoma Database Consortium, *NLR* neutrophil-to-lymphocyte ratio, *TKI* tyrosine kinase inhibitor, *IO* immuno-oncology therapy, *NIV* nivolumab, *PEM* pembrolizumab, *SIN* sintilimab, *TIS* tislelizumab, *TOR* toripalimab, *NA* not applicable, *IQR* interquartile range

The prognostic value of inflammatory index NLR has been investigated in RCC patients, and it is confirmed to be an independent prognostic factor. Inflammatory indices are considered as the most important endpoint in oncological studies, and the combination of NLR and the IMDC score demonstrates great application potential to reflect the heterogeneity of RCC [3]. Different from the IMDC score, which was established in the pre-immunotherapy era, the Meet-URO score is derived from a population treated with an immunotherapeutic strategy and better reflects the current treatment scenario [3, 4]. Compared to historical Meet-URO-related studies (Additional file 1), this small-size analysis explored the prognostic value of the Meet-URO score in similar disease status but with a different treatment type. Moreover, the results about the positive effect of nephrectomy are consistent with a subgroup analysis of the Meet-URO 15 study (HR = 0.48, 95% CI 0.33 to 0.69, *p* < 0.001) [5]. Although it didn’t reach statistical significance in multivariable analysis, more data based on a larger population is needed.

In conclusion, this study preliminarily illustrates that the Meet-URO score has the potential to present more accurate prognostic stratification than the IMDC score in mRCC patients receiving 2nd or 3rd-line TKIs-ICIs combination treatment. However, considering the limited number of participants and single-center design, more research based on larger cohorts is necessary to validate and strengthen these findings in the future.

### Supplementary Information

**Additional file 1: Table S1.** Summary of application of Meet-URO score in mRCC patients receiving systemic therapies.

**Additional file 2: Table S2.** Cox regression analysis for survival outcomes according to clinical features and the Meet-URO score.

## Data Availability

All data/materials in this present study are available from corresponding authors upon proper request.

## Abbreviations

TKI: Tyrosine kinase inhibitor
ICI: Immune checkpoint inhibitor
mRCC: Metastatic renal cell carcinoma
IMDC: International Metastatic RCC Database Consortium
NLR: Neutrophil-to-lymphocyte ratio
OS: Overall survival
CI: Confidence interval
HR: Hazard ratio
PFS: Progression-free survival
