# Supplementary material for: Validation of the Meet-URO score in metastatic clear cell renal cell carcinoma patients receiving second or third-line tyrosine kinase inhibitors-immune checkpoint inhibitors combination therapy
Source: J Transl Med. 2024 Mar 3;22:232. doi: 10.1186/s12967-024-05014-z (PMC10910860; doi:10.1186/s12967-024-05014-z)
Supplement: Supplementary file 1 — Additional file 1: Table S1. Summary of application of Meet-URO score in mRCC patients receiving systemic therapies. [file 12967_2024_5014_MOESM1_ESM.docx]

**Table S1** Summary of application of Meet-URO score in mRCC patients receiving systemic therapies

| Study | Cohort | Therapy line | Therapy type | Main findings |
| --- | --- | --- | --- | --- |
| Inflammatory indices and clinical factors in metastatic renal cell carcinoma patients treated with nivolumab: the development of a novel prognostic score (Meet-URO 15 study) | 571 patients collected retrospectively from 34 Italian centers | ≥ 2^nd^ line | IO (nivolumab) | The Meet-URO score allowed for the accurate stratification of pretreated mRCC patients receiving nivolumab and is easily applicable for clinical practice at no additional cost. |
| Validation of the Meet-URO score in patients with metastatic renal cell carcinoma receiving first-line nivolumab and ipilimumab in the Italian Expanded Access Program | 306 patients from the prospective Italian Expanded Access Programme | 1^st^ line | IO+IO (nivolumab + ipilimumab) | The Meet-URO score showed better prognostic classification than the IMDC alone in patients with mRCC at IMDC intermediate-poor risk treated with first-line nivolumab and ipilimumab (*c*-index: 0.73 *vs*. 0.65 [OS]; 0.67 *vs*. 0.59 [PFS]). |
| Application of the Meet-URO score to metastatic renal cell carcinoma patients treated with second- and third-line cabozantinib | 174 patients retrospectively collected from 10 centers | 2^nd^ and 3^rd^ | TKI (cabozantinib) | Meet-URO score provides a more accurate prognostic stratification than the IMDC score in mRCC patients treated with ⩾second-line cabozantinib besides nivolumab (*c*-index: 0.640 *vs*. 0.568 [OS]). |
| Prognostic stratification by the Meet-URO score in a real-world elderly population of patients (pts) with metastatic renal cell carcinoma (mRCC) receiving cabozantinib: A subanalysis of the prospective ZEBRA study (Meet-URO 9). | 104 patients from prospective Zebra study | 1^st^ -4^th^ | TKI (cabozantinib) | The Meet-URO score (using both original with 5 groups and modified with 3 groups) has shown a higher prognostic power than the IMDC score alone (*c*-index: 0.686 and 0.676 *vs*. 0.622 [OS]) |

*Abbreviations*: mRCC: metastatic renal cell carcinoma; IO: immuno-oncology therapy; TKI: tyrosine kinase inhibitor; IMDC: International mRCC Database Consortium; OS: overall survival; PFS: progression-free survival;
