# Supplementary material for: Validation of the Meet-URO score in metastatic clear cell renal cell carcinoma patients receiving second or third-line tyrosine kinase inhibitors-immune checkpoint inhibitors combination therapy
Source: J Transl Med. 2024 Mar 3;22:232. doi: 10.1186/s12967-024-05014-z (PMC10910860; doi:10.1186/s12967-024-05014-z)
Supplement: Supplementary file 2 — Additional file 2: Table S2. Cox regression analysis for survival outcomes according to clinical features and the Meet-URO score. [file 12967_2024_5014_MOESM2_ESM.docx]

**Table S2** Cox regression analysis for survival outcomes according to clinical features and the Meet-URO score

| Parameter | Univariable analysis | | | Multivariable analysis | | |
| --- | --- | --- | --- | --- | --- | --- |
|  | HR (95% CI) | *p*-value | | HR (95% CI) | *p*-value | |
| **Age** | 1.00 (0.95-1.06) | | 0.887 |  | |  |
| **Gender** |  | |  |  | |  |
| Female | 1.00 (Ref) | |  |  | |  |
| Male | 2.46 (0.32-18.61) | | 0.384 |  | |  |
| **ISUP grade** |  | |  |  | |  |
| <3 | 1.00 (Ref) | |  |  | |  |
| ≥3 | 0.99 (0.30-3.30) | | 0.992 |  | |  |
| **M stage** |  | |  |  | |  |
| Synchronous metastasis | 1.00 (Ref) | |  |  | |  |
| Metachronous metastasis | 1.51 (0.56-4.08) | | 0.413 |  | |  |
| **Meet-URO group** |  | |  |  | |  |
| Group 1 | 1.00 (Ref) | |  | 1.00 (Ref) | |  |
| Group 2 | 4.13 (1.31-13.01) | | **0.016** | 2.70 (0.77-9.52) | | 0.122 |
| Group 3 | 8.7 (2.32-32.63) | | **0.001** | 7.12 (1.79-28.39) | | **0.0054** |
| **N stage** |  | |  |  | |  |
| N0 | 1.00 (Ref) | |  |  | |  |
| N1 | 1.91 (0.51-7.10) | | 0.335 |  | |  |
| **Nephrectomy** |  | |  |  | |  |
| No | 1.00 (Ref) | |  | 1.00 (Ref) | |  |
| Yes | 0.26 (0.09-0.75) | | **0.013** | 0.43 (0.14-1.34) | | 0.1445 |
| **T stage** |  | |  |  | |  |
| <3 | 1.00 (Ref) | |  |  | |  |
| ≥3 | 1.31 (0.40-4.29) | | 0.659 |  | |  |

*Abbreviation:* HR: hazard ratio; CI: confidential interval; NLR: neutrophil-to-lymphocyte ratio; ISUP: International Society of Urological Pathology
